# From stroke workup to mitochondrial disease: A case report of MELAS

**DOI:** 10.1016/j.radcr.2025.09.031

**Published:** 2025-09-27

**Authors:** Yusuf Sevencan, Siddharth Rode, Ashley Park, Ilya Levin, Daniel Masri, Anna Derman

**Affiliations:** a Department of Radiology, Maimonides Medical Center, 4802 Tenth Avenue, Brooklyn, NY 11219, USA; b College of Medicine, State University of New York Downstate Health Sciences University, 450 Clarkson Avenue, Brooklyn, NY 11203, USA; c Department of Radiology, State University of New York Downstate Health Sciences University, 450 Clarkson Avenue, Brooklyn, NY 11203, USA

**Keywords:** MELAS syndrome, Mitochondrial disease, Stroke mimic, Diffusion-weighted MRI, Lactic acidosis, MT-TL1 gene mutation

## Abstract

Mitochondrial encephalomyopathy, lactic acidosis, and stroke-like episodes (MELAS) is a rare mitochondrial disorder that often presents with recurrent neurological deficits mimicking ischemic stroke. However, MELAS lesions characteristically violate vascular territories, a pattern that may be underrecognized in adult patients, particularly when vascular risk factors confound clinical suspicion. We present a case of a 36-year-old male with type 2 diabetes, tobacco use, and alcohol use disorder who experienced multiple recurrent stroke-like episodes involving the temporal and parietal lobes. Despite an extensive negative vascular and infectious workup, serial MRI demonstrated multifocal cortical and subcortical T2/FLAIR hyperintensities with restricted diffusion and evolving lesion patterns inconsistent with a vascular etiology. MR findings were suggestive of both cytotoxic and vasogenic edema, further supporting a metabolic cause. Genetic testing ultimately confirmed a heteroplasmic pathogenic variant in the MT-TL1 gene, consistent with MELAS. This case underscores the critical role of radiologic pattern recognition in diagnosing MELAS and the importance of distinguishing stroke-like lesions from true infarcts to guide appropriate clinical management.

## Introduction

Mitochondrial encephalomyopathy, lactic acidosis, and stroke-like episodes (MELAS) syndrome is a rare mitochondrial disorder characterized by multisystem involvement, predominantly affecting the nervous and muscular systems.

The hallmark clinical manifestation of MELAS is recurrent stroke-like episodes, often affecting the temporal, parietal, and occipital lobes, which can mimic ischemic infarcts, but do not conform to vascular territories. Radiologically, MELAS presents distinct imaging findings on MRI, such as cortical and subcortical T2/FLAIR hyperintensities, diffusion abnormalities, and increased lactate peaks on MR spectroscopy.

In this case report, we present a patient with MELAS who developed recurrent stroke-like episodes, with a focus on the imaging findings that provided key diagnostic clues. Understanding the radiologic features of MELAS is essential for early recognition and differentiation from other cerebrovascular pathologies, guiding appropriate management strategies.

## Case report

A 36-year-old male with a history of type 2 diabetes mellitus, tobacco use, and alcohol use disorder initially presented to the emergency department (Day 0) with dizziness, slurred speech, and blurry vision. Brain MRI revealed acute and subacute infarcts in the right temporoparietal region and the left anterior temporal lobe. An extensive stroke workup was initiated. Magnetic resonance angiography (MRA) and magnetic resonance venography (MRV) were unremarkable. Transthoracic and transesophageal echocardiography (TTE/TEE) showed no evidence of an embolic source. Cerebral angiography demonstrated only mild irregularity of the bilateral anterior cerebral arteries (ACA), with no evidence of vasculitis, aneurysm, or stenosis. Lumbar puncture (LP) was negative for HSV antigen, although HSV-1 IgG was positive. He completed a 3-week course of acyclovir for possible HSV encephalitis, given the bilateral temporal lobe involvement, and was discharged home on dual antiplatelet therapy (DAPT) with plans for loop recorder placement.

**Fig 1:**
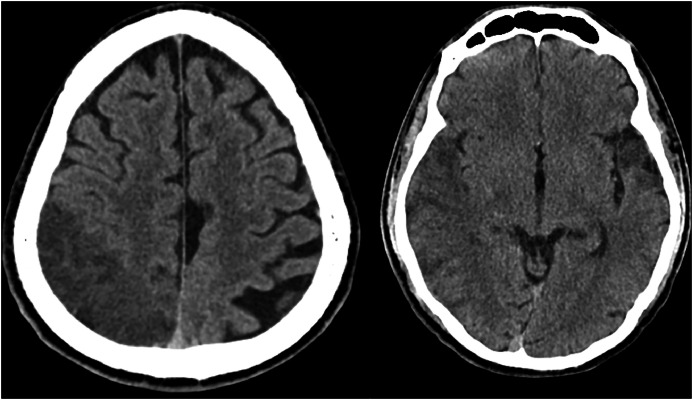
Non-contrast head CT shows loss of gray-white matter differentiation and sulcal effacement in the right MCA and posterior watershed territories, consistent with cytotoxic edema.

**Fig 2:**
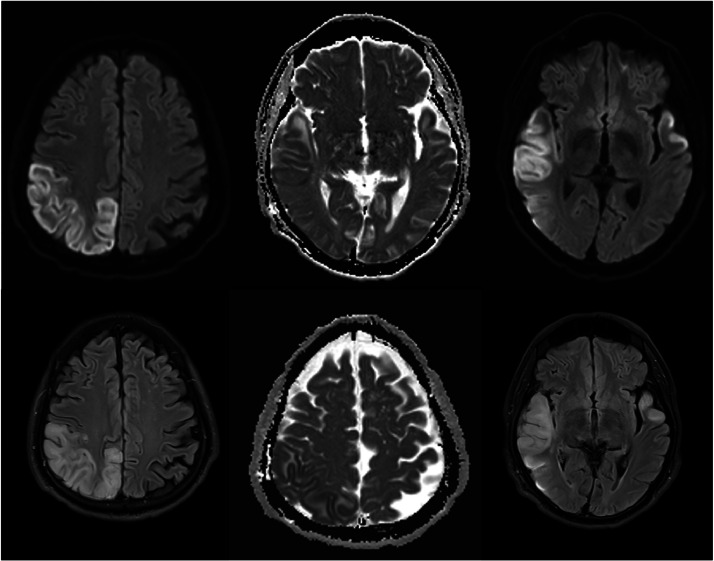
MRI brain (Left to right: DWI, ADC, and FLAIR sequences) demonstrates multifocal areas of cortical based restricted diffusion and FLAIR hyperintensity involving the right MCA/posterior PCA/ACA territories and left anterior temporal lobe.

On Day 21, the patient re-presented with left arm weakness and tingling, left facial droop, and gait instability after a fall. On exam, he exhibited left-sided visual and tactile neglect, although strength, sensation, and mentation were otherwise intact. Non-contrast CT head revealed infarcts in the right middle cerebral artery (MCA) and posterior watershed territories, as well as a small left anterior temporal infarct (Fig. 1). MRI confirmed acute on subacute infarcts in these same regions (Fig. 2). Repeat LP and urine toxicology were negative. Laboratory studies were unremarkable except for mildly elevated ESR (18) and persistently positive HSV-1 IgG. C-reactive protein (CRP), creatine phosphokinase (CPK), folate, LDL, A1C, HIV, RPR, and hepatitis panels were all within normal limits. EEG revealed right temporoparietal delta slowing, consistent with focal cerebral dysfunction.

Given the bilateral, multifocal strokes and unrevealing vascular and infectious workup, differential considerations expanded to include embolic phenomena, hypercoagulable states, systemic inflammatory disease, and mitochondrial encephalopathies such as adult-onset MELAS. Autoimmune serologies and metabolic evaluations were initiated.

On Day 35, the patient was found on the floor after an apparent seizure, described as left arm clonic activity followed by loss of consciousness. In the ED, he was post-ictal and confused. Physical examination was notable for new-onset bilateral hearing loss. There were no signs of trauma, tongue laceration, or incontinence. Serum lactate was elevated at 5.5 mmol/L, and LP was repeated. EEG again showed no epileptiform activity. The patient was started on levetiracetam (Keppra).

**Fig 3:**
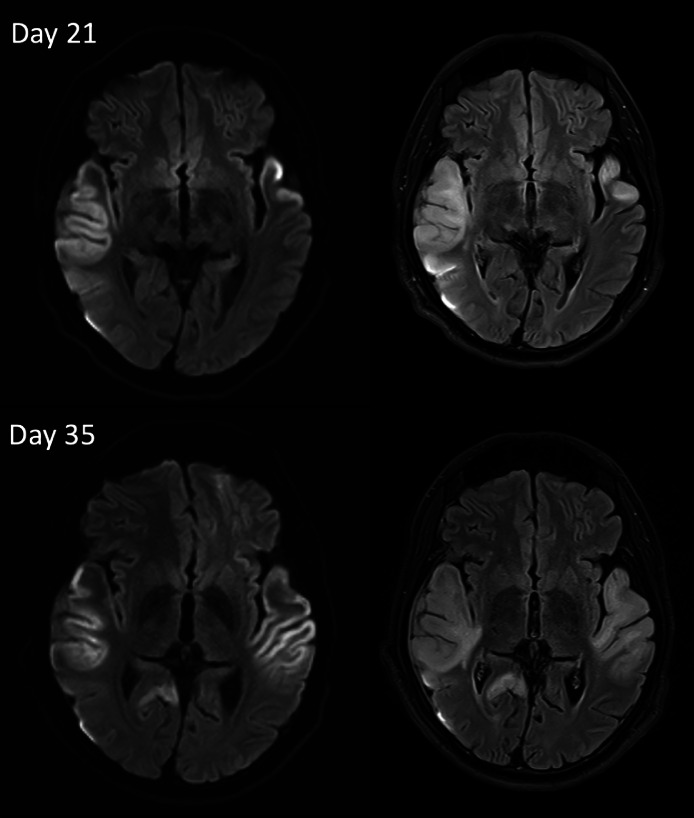
Diffusion restriction and FLAIR images from Day 21 and Day 35 show worsening cortical based diffusion restriction in the left temporal lobe in combination with subcortical edema and component of new cortical based diffusion restriction in the right posterior temporal occipital PCA territory.

**Fig. 4:**
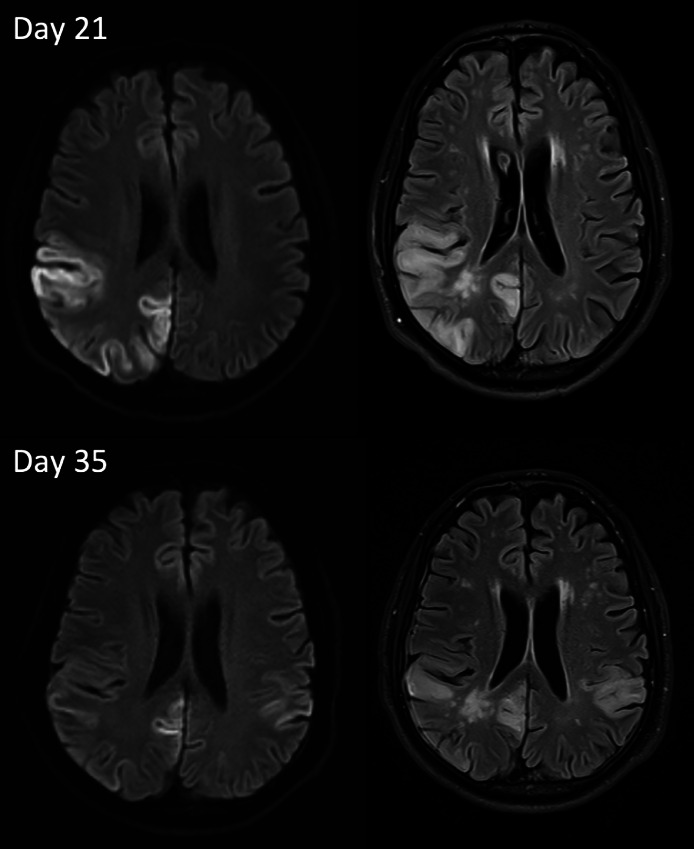
Diffusion restriction and FLAIR images from Day 21 and Day 35 show right MCA and PCA territory cortical based diffusion restriction, not corresponding to a single vessel disease and suggestive of possible nonvascular source.

Repeat brain MRI demonstrated interval progression of signal abnormalities in the left temporal and adjacent parietal cortex, with new cortical restricted diffusion and extension into the underlying subcortical white matter, suggesting a combination of cytotoxic and vasogenic edema. Comparative diffusion restriction and FLAIR sequences from Day 21 and Day 35 (Fig 3, Fig. 4) illustrate worsening cortical-based diffusion restriction in the left temporal lobe with associated subcortical edema, as well as new cortical involvement in the right posterior temporal–occipital PCA territory and right MCA/PCA regions, findings not confined to a single vascular distribution and raising suspicion for a nonvascular source such as MELAS.

## Discussion

MELAS syndrome is a rare mitochondrial disorder characterized by multisystem involvement, predominantly affecting the nervous and muscular systems. It is caused by pathogenic mutations in mitochondrial DNA (mtDNA), with the most common mutation being A3243G in the MT-TL1 gene, which encodes mitochondrial tRNA-Leu(UUR) [1]. This mutation leads to defective mitochondrial energy metabolism, resulting in neuronal dysfunction and metabolic crises.

The hallmark clinical manifestation of MELAS is recurrent stroke-like episodes. Other neurological symptoms include seizures, dementia, headaches, altered mental status, cortical visual loss, and motor deficits. Age of initial presentation is typically under 40 years [1].

MELAS syndrome is radiologically characterized by multifocal stroke-like lesions that violate vascular territories, most often involving the temporal, parietal, and occipital lobes [2,3]. These lesions typically appear hyperintense on T2-weighted and FLAIR MRI sequences and may exhibit variable diffusion restriction on DWI/ADC due to mixed cytotoxic and vasogenic edema. The co-existence of cytotoxic and vasogenic edema helps distinguish MELAS from vascular strokes, as vasogenic edema does not typically present until days after an ischemic stroke. In MELAS, by contrast, abnormal mitochondria in vascular endothelial and smooth muscle cells may impair autoregulatory mechanisms, producing early blood–brain barrier dysfunction and vasogenic edema alongside the neuronal energy failure responsible for cytotoxic edema [6]. Additionally, perfusion-weighted imaging (PWI) generally lacks the perfusion deficits seen in ischemic infarcts, further differentiating MELAS from vascular strokes.

Stroke-like lesions may show mass effect or contrast enhancement in the subacute phase due to blood-brain barrier disruption or microvascular reperfusion due to autoregulatory impairment. Over time, these lesions often regress with clinical improvement but can leave residual parenchymal atrophy [3]. Proton MR spectroscopy (MRS) provides additional diagnostic value by demonstrating elevated lactate peaks, a hallmark of mitochondrial dysfunction [4].

While these imaging patterns are varied, 1 particular feature is especially diagnostic. A key radiologic teaching point in MELAS is the striking cortical-based diffusion restriction that often spans multiple vascular territories, a hallmark that diverges from typical ischemic infarcts. This cortical predilection is usually accompanied by vasogenic edema in the adjacent subcortical white matter, with relative sparing of the deep white matter, a pattern highly atypical for embolic or thrombotic strokes. The preferential involvement of cortical grey matter in MELAS reflects its greater mitochondrial density (over 50% more than white matter) and higher metabolic demand, making it more susceptible to energy failure during mitochondrial dysfunction [7]. In contrast, deep white matter, composed largely of myelinated axons, has lower baseline metabolic activity and greater tolerance to metabolic stress, accounting for its relative preservation in MELAS episodes.

Furthermore, in ischemic infarcts due to large-vessel occlusion, the territorial distribution of restricted diffusion corresponds precisely to the vascular supply of a single artery, such as the MCA or PCA. These infarcts often extend deeply into the subcortical and periventricular white matter, following a wedge-shaped or linear pattern that conforms to arterial branching. In contrast, MELAS lesions often cross arterial boundaries, exhibit patchy, migratory involvement, and lack the perfusion deficits typically seen on perfusion imaging, further distinguishing them from classic infarcts. Recognizing this metabolism-driven, cortex-predominant, non-vascular distribution on MRI is critical for raising early suspicion for MELAS and prompting appropriate metabolic and genetic evaluation.

A key distinguishing feature of MELAS is its relapsing-remitting nature, where lesions may resolve, recur, or migrate to different brain regions with subsequent episodes. This dynamic "shifting" pattern of lesion involvement is thought to be driven by metabolic crises rather than thromboembolic events, which typically result in fixed infarcts. The migratory nature of MELAS lesions underscores its metabolic basis and differentiates it from ischemic strokes, where infarcts remain static and confined to vascular distributions.

Beyond the central nervous system, MELAS also affects skeletal muscles, with muscle biopsies revealing ragged red fibers, a sign of mitochondrial dysfunction. Electron microscopy further demonstrates abnormal mitochondria, providing additional diagnostic confirmation in suspected cases. The combination of these characteristic radiologic findings and histopathological evidence is crucial in differentiating MELAS from other cerebrovascular, metabolic, and inflammatory conditions, guiding early diagnosis and management.

Management of MELAS remains largely symptomatic, with a focus on metabolic support and stroke prevention strategies. L-arginine and citrulline supplementation have shown promise in mitigating stroke-like episodes by improving nitric oxide availability and cerebral perfusion. Additionally, mitochondrial-targeted therapies such as coenzyme Q10, riboflavin, and creatine supplementation have been explored to enhance mitochondrial function [5].

Radiologists play a pivotal role in the early recognition of MELAS, particularly in differentiating it from ischemic strokes and other metabolic or inflammatory disorders. Serial imaging is often required to track lesion evolution and monitor disease progression. Increased awareness of these imaging findings can aid in early diagnosis and management, ultimately improving patient outcomes.

## Patient consent

Necessary written, informed consent has been obtained from the patient for use of materials in this case report.
